# AI for pathologists: a universal lymph node metastasis detection app that enhances efficiency while preserving diagnostic accuracy

**DOI:** 10.1002/2056-4538.70073

**Published:** 2026-01-20

**Authors:** Jennifer Vazzano, Bindu Challa, Vidya Arole, Konstantin Shilo, Sarah Reuss, Peter Kobalka, Swati Satturwar, Juan Xie, Dongjun Chung, Saba Shafi, David Kellough, Erin Palermini, Zaibo Li, Wei Chen, Anil Parwani, Shaoli Sun

**Affiliations:** ^1^ Department of Pathology The Ohio State University Wexner Medical Center Columbus OH USA; ^2^ The Interdisciplinary Ph.D. Program in Biostatistics The Ohio State University Columbus OH USA; ^3^ Department of Biomedical Informatics The Ohio State University Columbus OH USA

**Keywords:** artificial intelligence (AI), digital pathology, lymph node metastasis, image analysis, cancer diagnostics, computational pathology, diagnostic efficiency

## Abstract

Increasing workload combined with the shortage of pathologists is the leading cause of diagnostic errors and delays. Nonetheless, in clinical practice, pathologists often spend hours on tedious tasks such as counting mitoses and searching for lymph node micro‐metastasis, which may yield unreliable results. The advent of digital pathology and the development of artificial intelligence (AI) applications (app) for image analysis have opened new possibilities for improving the efficiency and accuracy of pathologists. However, the perceived black box nature of AI has led to skepticism among many pathologists about its diagnostic capabilities, resulting in a lack of trust in AI. In addition, it is a common belief that AI applications should be limited to the areas they were trained in, which has significantly limited their generalizability. Given the homogeneous cell population of lymph nodes and overlapping of tumor morphology across different organs, we hypothesized that a lymph node metastasis detection application trained on a few organs could potentially recognize metastasis from multiple organs. We used the commercially available Visiopharm app (AI tool), initially trained on lymph node metastases from breast and colon cancer, to detect metastasis of 12 distinct types of cancer from 15 organ systems based on the analysis of 172 slides (all with corresponding immunohistochemical staining confirmation). Furthermore, by using the annotation map generated by the app as a guide, pathologists were also able to reduce the time spent searching for metastasis substantially (from 54.7 to 42.1 s per slide on average) without compromising diagnostic accuracy. With pathologists serving as the trusted gatekeepers and the development of more sophisticated image analysis applications, the use of AI can help to address the shortage of pathologists, enhance their performance and eventually improve patient care.

## Introduction

Pathologists play an essential role throughout the continuum of medical care, from prenatal diagnosis to postmortem examination. Their work is essential for diagnosing diseases, which is the basis for all treatments and prognoses, especially in cancer. It is estimated that approximately 40% of men and women worldwide will be diagnosed with cancer at some point in their lives [1].

From 2007 to 2017, the number of pathologists in the USA decreased by 18% and their workload increased by 42% [2, 3]. A similar trend has been observed in the UK, where it is estimated that a 45% increase in staff is needed to meet Health Education England's goal of providing quality care for cancer patients by 2029 [4]. The shortage is particularly pronounced in developing countries [5]. Factors contributing to the increased workload include an aging population leading to more cancer diagnoses and the requirements of precision treatment [6].

The combination of pathologist shortage with increasing workload could inevitably lead to delays and increased errors in diagnosis [2]. Meanwhile, in clinical practice, pathologists spend a substantial amount of time on repetitive tasks such as searching for metastases and counting mitosis manually. Overlooking microscopic metastatic foci is common and can result in inaccurate staging and/or prognosis which in turn can lead to inaccurate therapy (i.e. for colorectal, gastric, cervical, endometrial, and vulvar carcinoma) [7, 8, 9, 10]. Factors contributing to this suboptimal performance include increasing workload, lack of experience, visual fatigue, and limitations of human vision [11, 12, 13, 14]. Many newly developed apps designed to identify micro‐metastases [15, 16] have shown promising results. However, most of these apps were trained on a single organ and were recommended for use only for that organ, which severely limits their utility. In addition, due to the ‘black box’ nature of deep learning in the development of the apps, many pathologists lack the confidence to rely on artificial intelligence (AI) for accurate diagnoses [17].

Given the homogeneous cell population in lymph nodes and the overlapping tumor morphology from a variety of organs, we hypothesize that an app trained on one organ can potentially also recognize tumor metastases from different organs. We designed this study by using the Visiopharm Metastasis Detection AI, which was trained on lymph node metastases from both the breast and colon, to test whether this app can recognize other tumor types from organs that were not included in the training.

## Materials and methods

We conducted a retrospective search of our Laboratory Information System to identify tumor cases with either positive or negative lymph nodes with corresponding immunohistochemical (IHC) staining confirmation. A total of 172 consecutive lymph node slides were selected from 78 total cases; some slides were from the same case and, in total, there were 69 positive and 103 negative slides. We excluded slides with inadequate nodal tissue (<1 mm^2^), artifacts, necrosis, poor tissue staining, cases with equivocal diagnoses, and cytology slides. All slides were de‐identified to prevent any bias. Institutional Review Board (IRB) approval was not required for this study because it involved a retrospective case series with fully de‐identified data and did not meet the definition of human subjects research, per [NCH and CHOP's] IRB policy and 45 CFR 46. The study was conducted in accordance with institutional policy and applicable regulations.

**Figure 1:**
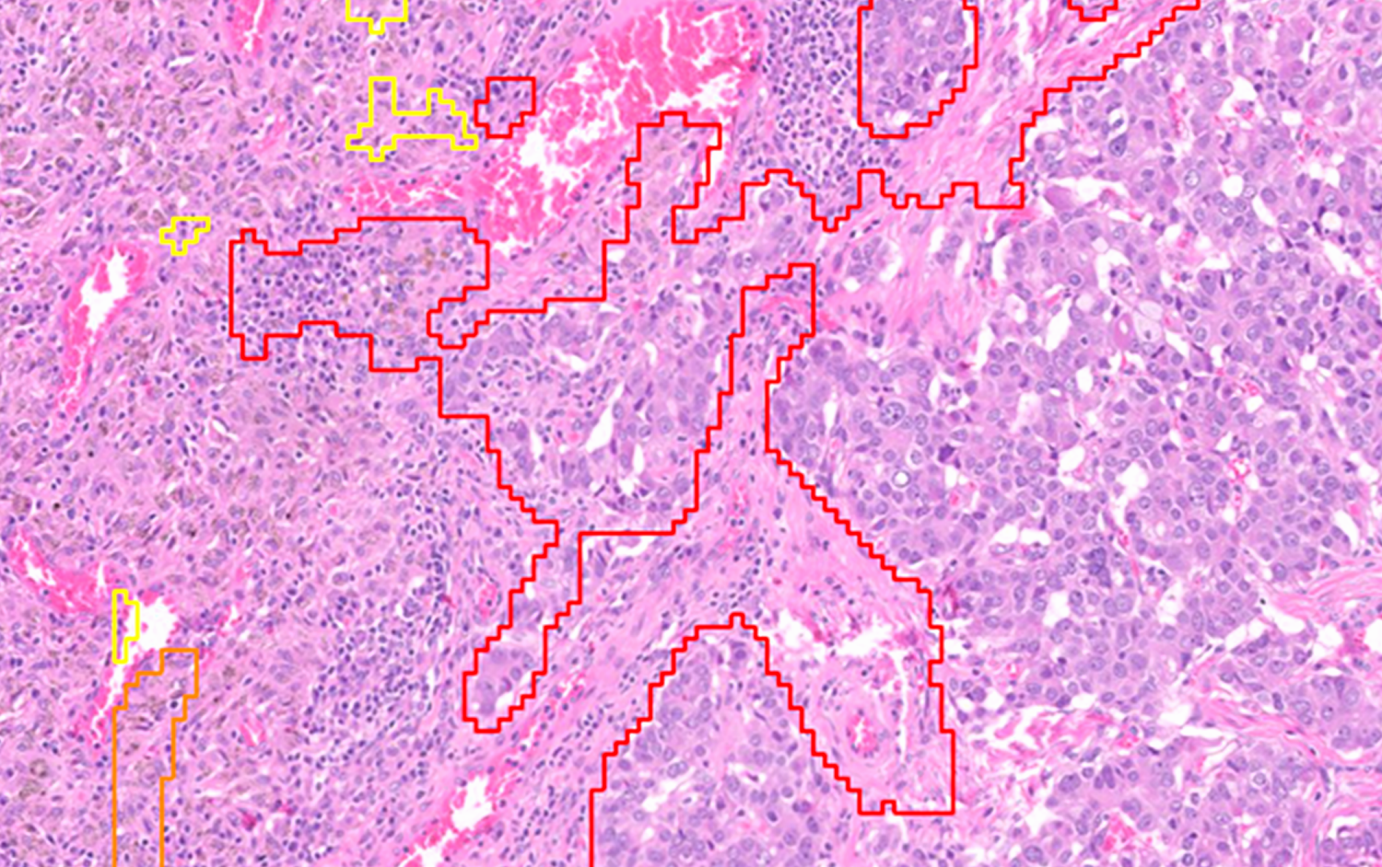
Example histology slide with annotations for all three AI colors (yellow, orange, and red).

We used a commercially available ‘Metastasis Detection AI’ from Visiopharm to create an AI map for every slide. The AI was initially trained by the Visiopharm development team on lymph node metastases from both breast and colon cancers, with 300 cases for each. This AI algorithm included tissue detection, metastasis detection, and post‐processing to generate a three‐colored annotation map based on the probability of cancer presence (red color = high probability, orange color = intermediate probability, and yellow color = low probability). If no labeling was present, it indicated that there was no tumor present. An example histology slide with all three AI colors annotated is presented in Figure 1.

Six pathologists participated in this study, with each performing three runs of reading for each case (Table 1). The three runs included reviewing hematoxylin and eosin (H&E) staining only, followed by reviewing H&E with AI map (H&E with AI), and then reviewing H&E with IHC staining (H&E with IHC). Pathologists recorded their diagnosis (yes/no for tumor) and the time spent on the diagnosis for each slide in each run. A 2‐week washout interval between each run was mandated to erase the pathologists' memory of their diagnoses from the previous runs. The ground truth for each slide was established based on a combination of reviewing the H&E, AI map, and IHC stain.

**Table 1:** A total of six pathologists/trainees participated in the study

Pathologist ID	Years of training/practice	Title	Subspecialty
P1	29	Attending	GI
P2	24	Attending	Thoracic
P3	11	Attending	Community
P4	11	Attending	Neuropathology and cytology
P5	7	Attending	GU and cytology
P6	4	Resident	General

**Figure 2:**
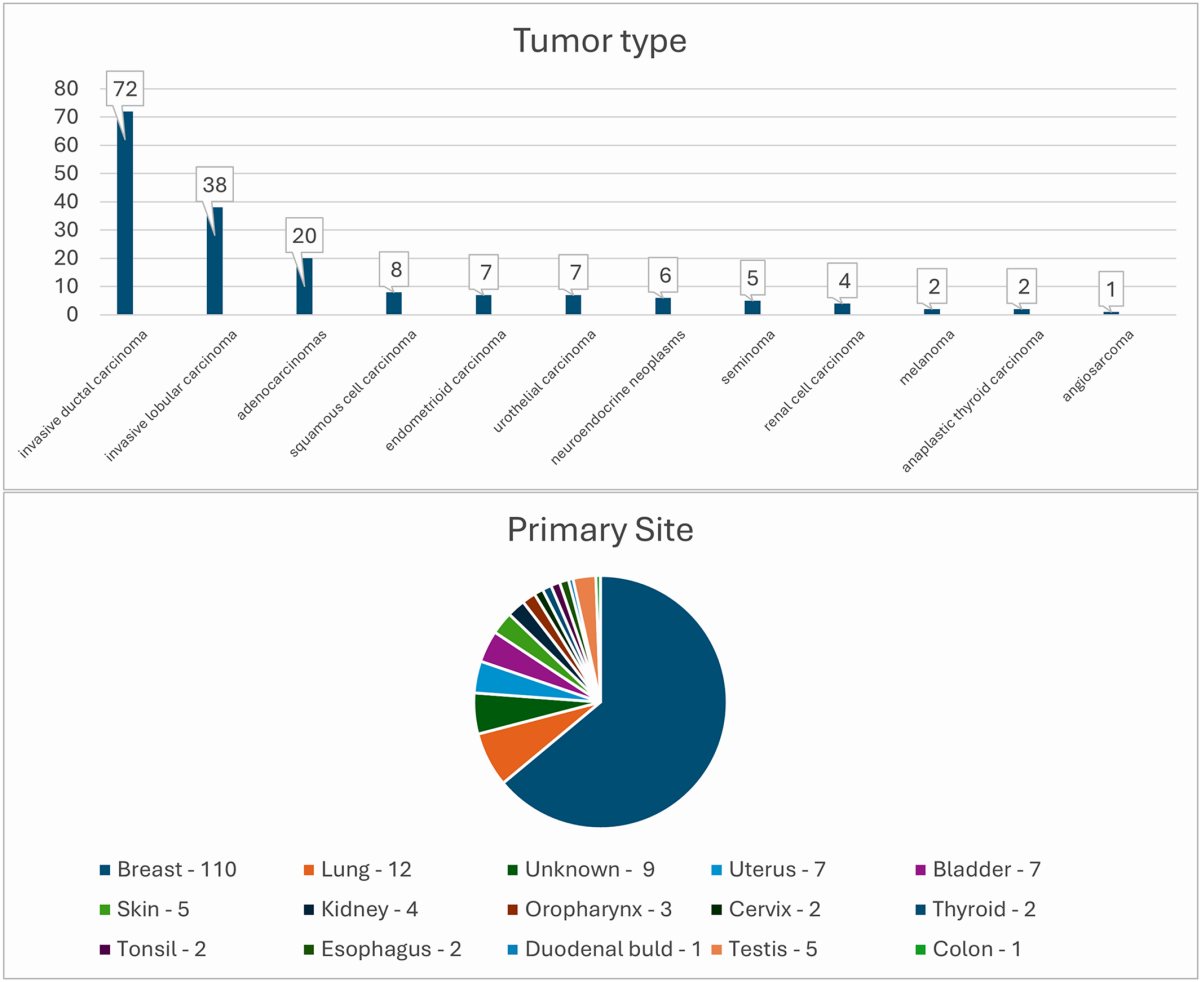
Bar chart for tumor types and pie chart for tumor primary sites.

For this study, we selected 12 distinct types of tumors originating from 15 different organs. The adenocarcinomas included lung adenocarcinomas, a colon adenocarcinoma, gastroesophageal adenocarcinomas, and a duodenal bulb adenocarcinoma. The neuroendocrine neoplasms included lung small cell carcinoma, a typical carcinoid tumor, and poorly differentiated neuroendocrine carcinoma. Figure 2 presents the types of tumors, their corresponding frequencies, and the organ systems from which the cancers originated, with invasive ductal carcinoma of the breast being the most common tumor type and breast being the most common primary site.

In this study, a combination of H&E, AI, and IHC staining confirmation served as the gold standard. For each pathologist and diagnostic modality, we constructed a 2 × 2 contingency table to record the number of true positives (TPs), true negatives (TNs), false positives (FPs), and false negatives (FNs).

Sensitivity [TP/(TP + FN)], specificity [TN/(TN + FP)], and accuracy [(TP + TN)/total cases] were calculated per pathologist. Each pathologist's results were computed separately (*n* = 172 slides per pathologist), and summary statistics (mean and median) across the six pathologists were reported. Wilcoxon signed rank tests with Bonferroni correction were used for statistical analysis for multiple tests (reading time, accuracy, sensitivity, and specificity).

## Results

### Comparison of the performances among reading by H&E only, H&E with AI, and H&E with IHC

#### Reading time

**Figure 3:**
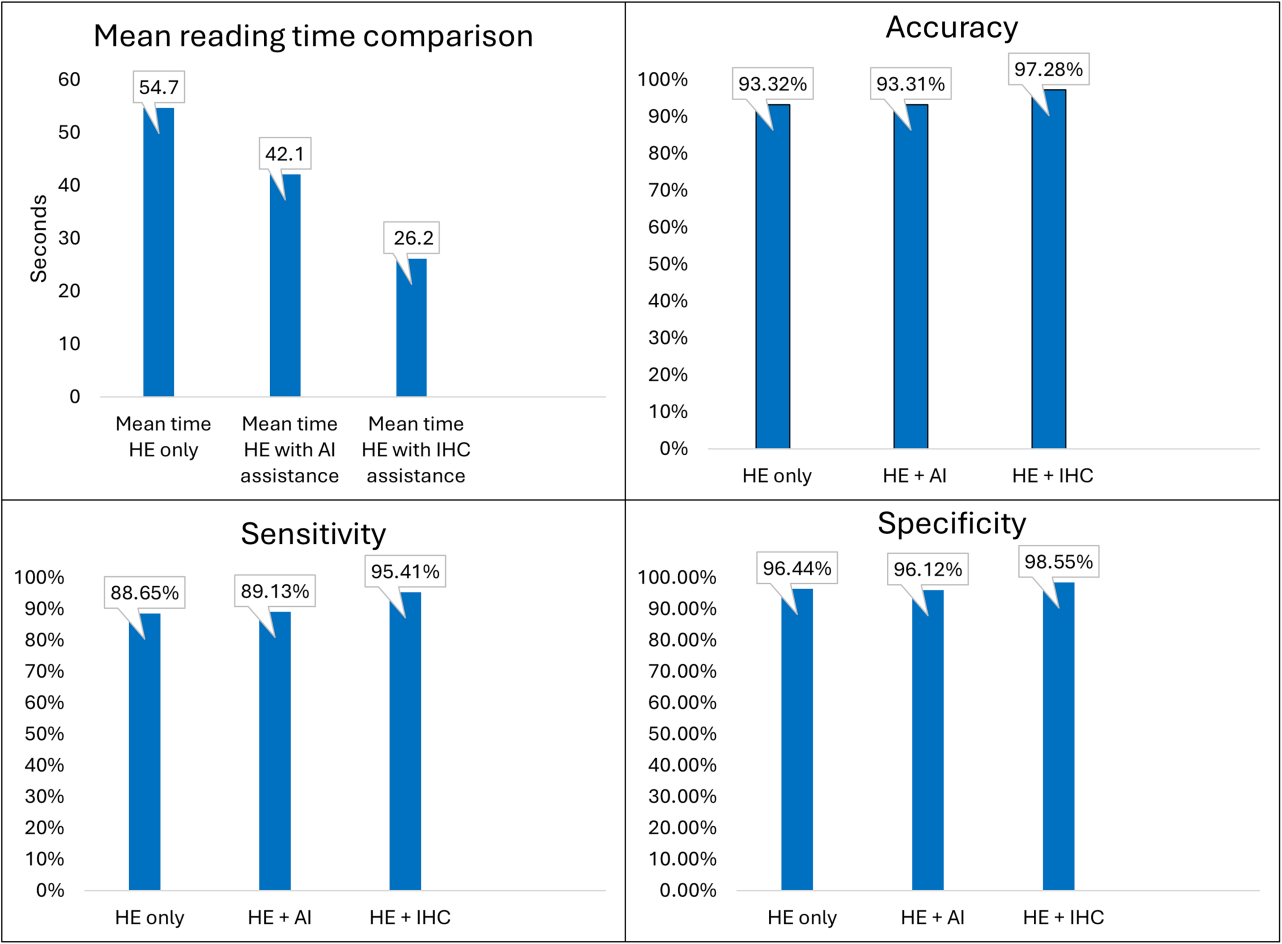
Average reading times and accuracy, sensitivity, and specificity comparisons.

We used Q1 (25%) and Q3 (75%) to report the range for reading time. The time taken to evaluate H&E only slide ranged from 16.4 to 162.7 s, with a mean of 54.7 s (see Table 2). The time taken to read the H&E slide with AI assistance ranged from 13.33 to 168.48 s, with a mean of 42.1 s. The time taken to read the H&E slide with IHC assistance ranged from 4.78 to 88.29 s, with a mean of 26.2 s. On average, the evaluation time decreased from 54.7 to 42.1 s with AI assistance (Figure 3). However, with a mean reading time of 26.2 s, H&E with IHC staining is still more efficient, despite its associated cost and longer turnaround time. There was a statistically significant difference for reading time between H&E versus H&E with AI (adjusted *p* value = 2.46E‐44), H&E versus H&E with IHC (adjusted *p* value = 2.40E‐75), and H&E with IHC versus H&E with AI (adjusted *p* value = 1.09E‐33) (Table 4).

**Table 2:** Mean and median reading times (unit: seconds)

Technology	Mean	Median
H&E only	54.7	30
H&E with AI	42.1	19
H&E with IHC	26.2	10

**Table 3:** Mean and median of accuracy, sensitivity, and specificity

Modality	Sensitivity mean (%)	Sensitivity median (%)	Specificity mean (%)	Specificity median (%)	Accuracy mean (%)	Accuracy median (%)
H&E only	88.65	89.14	96.44	98.55	93.32	94.19
H&E with AI	89.13	91.30	96.12	97.58	93.31	93.90
H&E with IHC	95.41	95.65	98.55	99.03	97.28	97.09

#### Accuracy

Out of the total 172 cases, the number of misdiagnoses for H&E stain only ranged from 8 to 20, with a mean of 11.5 misdiagnoses (accuracy 93.32%) (Table 3 and Figure 3). With AI assistance, the number of misread cases ranged from 6 to 18, with a mean of 11.6 misdiagnoses (accuracy 93.31%). With IHC staining, the number of misread cases ranged from 4 to 6, with a mean of 4.6 misdiagnoses (accuracy 97.28%).

There was no significant difference between H&E only and H&E + AI (adjusted *p* value = 1), between H&E only and H&E + IHC (adjusted *p* value = 0.10656), or between H&E + IHC and H&E + AI (adjusted *p* value = 0.10656) (Table 4).

#### Sensitivity

Sensitivity mean for H&E only was 88.65% (85.7–89.1%). Sensitivity mean for H&E + AI was 89.13% (86.59–92.39%). Sensitivity mean for H&E + IHC was 95.41% (94.57–96.74%) (Table 3 and Figure 3). There was no significant difference between H&E only and H&E + AI (adjusted *p* value = 1), between H&E only and H&E + IHC (adjusted *p* value = 0.10503), or between H&E + IHC and H&E + AI (adjusted *p* value = 0.09375) (Table 4).

#### Specificity

Specificity mean for H&E only was 96.44% (96.1–100%). Specificity mean for H&E + AI was 96.12% (95.63–98.79%). Specificity mean for H&E + IHC was 98.55% (97.57–99.03%) (Table 3 and Figure 3). There was no statistically significant difference between H&E only and H&E + AI, H&E only and H&E + IHC (adjusted *p* values = 1), or between H&E + IHC and H&E + AI (adjusted *p* values = 0.4086) (Table 4).

After correcting for multiple testing, the reading time for the three pairs of comparisons was all significantly different, but the accuracy, sensitivity, and specificity are comparable (Table 4). Overall, our results demonstrate that the AI assistance of the lymph node metastasis detection app can not only recognize a variety of tumor types but also save pathologists' time without compromising the accuracy of their diagnosis.

**Table 4:** Bonferroni‐adjusted *p* values for comparisons between diagnostic modalities

Metric	H&E versus H&E + AI	H&E versus H&E + IHC	H&E + IHC versus H&E + AI
Time	2.46 × 10^−44^	2.40 × 10^−75^	1.09 × 10^−33^
Accuracy	1	0.10656	0.10656
Sensitivity	1	0.10503	0.09375
Specificity	1	1	0.4086

## Discussion

The advent of AI has significantly advanced several fields, including digital pathology. Our study highlights the potential use of AI as a screening tool for detecting lymph node metastasis from multiple organ systems. It shows that an app trained to detect lymph node metastasis in tumors from only two organs can also recognize tumors from multiple other organ systems, supporting that it could be used to detect lymph node metastasis more universally.

AI's high sensitivity has proven beneficial and can even improve IHC staining interpretation. This high sensitivity supports the potential role of AI as a screening tool. High false‐positive rates do not typically pose a challenge for experienced pathologists. With their extensive training, pathologists can reliably distinguish between tumor cells and non‐tumor cells such as crushed lymphoid cells and macrophages on H&E slides. For more challenging cases, IHC can be employed to confirm the diagnosis. AI also has the potential to reduce the number of IHC orders, which could lead to cost savings and improving turnaround times [18].

The ‘black box’ nature of AI can indeed make pathologists and other medical professionals skeptical about its usability [17]. However, it is important to note that the learning process of AI aligns well with the principles of professional medical practice. Both are based on the accumulation of recognition, knowledge, interpretation and correlation. AI, built on similar principles, learns and improves its diagnostic skill by training with massive data to fine‐tune its performance. As we continue to demystify the ‘black box’ of AI and improve its interpretability, we can expect its acceptance and utility in the medical field to grow more rapidly.

At this stage of AI development, the presence of pathologists as gatekeepers is crucial to ensure accurate diagnosis. Concerns regarding AI replacing pathologists are not supported by current evidence. Instead, AI can serve as an effective adjunct or assistive tool to enhance the performance of pathologists. It can assist in screening, reducing the number of IHC orders, and improving turnaround times, thereby enhancing the overall efficiency of the pathology workflow, ultimately leading to better patient care with less cost [19, 20].

We have validated this universal lymph node metastasis detection app and are ready for its implementation in our digital workflow. This application aims to improve the efficiency of pathologists. Following the completion of the current study, we conducted a subsequent prospective study to further corroborate our findings. In this subsequent prospective study of 60 clinical cases, AI detected two positive cases that would have been missed by pathologists, even with IHC staining (unpublished observations). These findings support its potential value in clinical practice.

In conclusion, we have demonstrated that this app can potentially work as a valuable universal screening tool for lymph node metastasis in multiple organs, and integrating AI into digital pathology workflow can significantly enhance the accuracy and efficiency of pathologists. Continued development of AI is expected to further improve efficiency and diagnostic precision in digital pathology. AI represents a valuable adjunct to the work of pathologists and warrants further integration into routine workflows.

## Author contributions statement

ShS was responsible for the conception and design with help from ZL, WC and AP. Material preparation and data collection were performed by JV, ShS, DK, EP, SaS, and BC. JV, KS, SR, PK, SwS, and ShS performed microscopic review of the slides. DC and JX performed statistical analysis. The final draft of the manuscript was written by JV, and all authors commented on previous versions of the manuscript. All authors read and approved the final manuscript.

## Data Availability

The datasets generated during and/or analyzed during the current study are available from the corresponding author on reasonable request.
